# Analysis of the competitiveness and complementarity of China-Vietnam bilateral agricultural commodity trade

**DOI:** 10.1371/journal.pone.0302630

**Published:** 2024-04-25

**Authors:** Jinjin Tian, Yulin Zhu, Thi Bich Nhi Hoang, Benjamin Kofi Tawiah Edjah

**Affiliations:** 1 International College, Central South University of Forestry and Technology, Changsha, China; 2 College of Economics, Central South University of Forestry and Technology, Changsha, China; 3 School of Economics and Management, Wuhan University, Wuhan, China; Mahidol University International College, THAILAND

## Abstract

Vietnam’s agricultural exports to China have remained strong, with the country maintaining its position as the top destination for Agri-products. This article primarily utilizes the Revealed Comparative Advantage (RCA) Index, and Trade Complementarity (TC) index to examine the trade comparative advantage, and the complementary of twenty major agricultural products between China and Vietnam from 2012 to 2021. The study results showed that Vietnam and China frequently exchange agricultural products. Vietnam has more stronger competitiveness than China in terms of agricultural products. China’s exports to Vietnam were highly complementary to Vietnam’s imports in category 0 whiles Vietnam’s exports to China showed strong complementarity with China’s imports in category 2. This paper analyzes the complementarity and comparative advantages of agricultural trade between China and Vietnam, and proposes informed suggestions for policy-making to promote agricultural trade between the countries. The proposed suggestions aim to expand agricultural trade between the two countries, reduce the trade imbalance, and achieve mutual benefit and win-win results.

## 1. Introduction

Agriculture serves as the backbone of economies worldwide, providing sustenance, income, and employment opportunities to millions. Its significance transcends mere sustenance, driving economic growth and fostering socio-economic development. The World Bank underscores the pivotal role of agriculture in enhancing prosperity, citing its potential to generate income up to four times more efficiently than other industries. With approximately 4% of the global GDP and even higher proportions in developing nations like Burkina Faso, Rwanda, and Ethiopia, it forms the cornerstone of many economies [1]. Moreover according to OECD on it study of the “Economic Importance of Agriculture for Poverty Reduction,” 2010 [2], reveals that agricultural growth has played a significant role in reducing poverty by creating jobs, boosting incomes, and lowering food prices. Countries that have prioritized agricultural development have experienced impressive progress in poverty reduction. These countries have invested heavily in agricultural research, extension, and education, leading to increased productivity and competitiveness. Additionally, favourable trade policies have contributed to the success of these countries in reducing poverty.

Against these development, it becomes imperative to delve into the dynamics of agricultural trade, particularly between nations of strategic importance. China and Vietnam exemplify such nations, where bilateral agricultural trade has witnessed remarkable growth. Their cross-border trade primarily operates through sub-border gateways, facilitated by their extensive 1,450-kilometer shared border spanning seven Vietnamese provinces and two Chinese provinces. The establishment of twenty-eight "border gate economic zones" along this border further catalyzes trade activities. With China agriculture plays a crucial role in its economic development. It contributes to the national economy through production, market, factor, and foreign exchange earning contributions. It remains an essential driving force for the growth of other sectors like industry, transportation, construction, and services. Excessive taxation on agriculture and strict policies on rural-urban labor movement hindered agricultural growth before 1978, but economic reforms since then have improved the situation [3].

Moreover, according to the Vietnam government, the implementation of the Regional Comprehensive Economic Partnership (RCEP) on January 1, 2022, promises to bolster economic cooperation between China and Vietnam. In the aftermath, China has pledged support to Vietnam’s agricultural exports, signalling potential growth opportunities in bilateral trade. Notably, Vietnam’s agricultural exports to China surged by 12.41% to $8.886 billion in 2021, with China emerging as a vital market for Vietnamese fruits, particularly accounting for 65% of Vietnam’s fruit exports [4].

**Fig 1:**
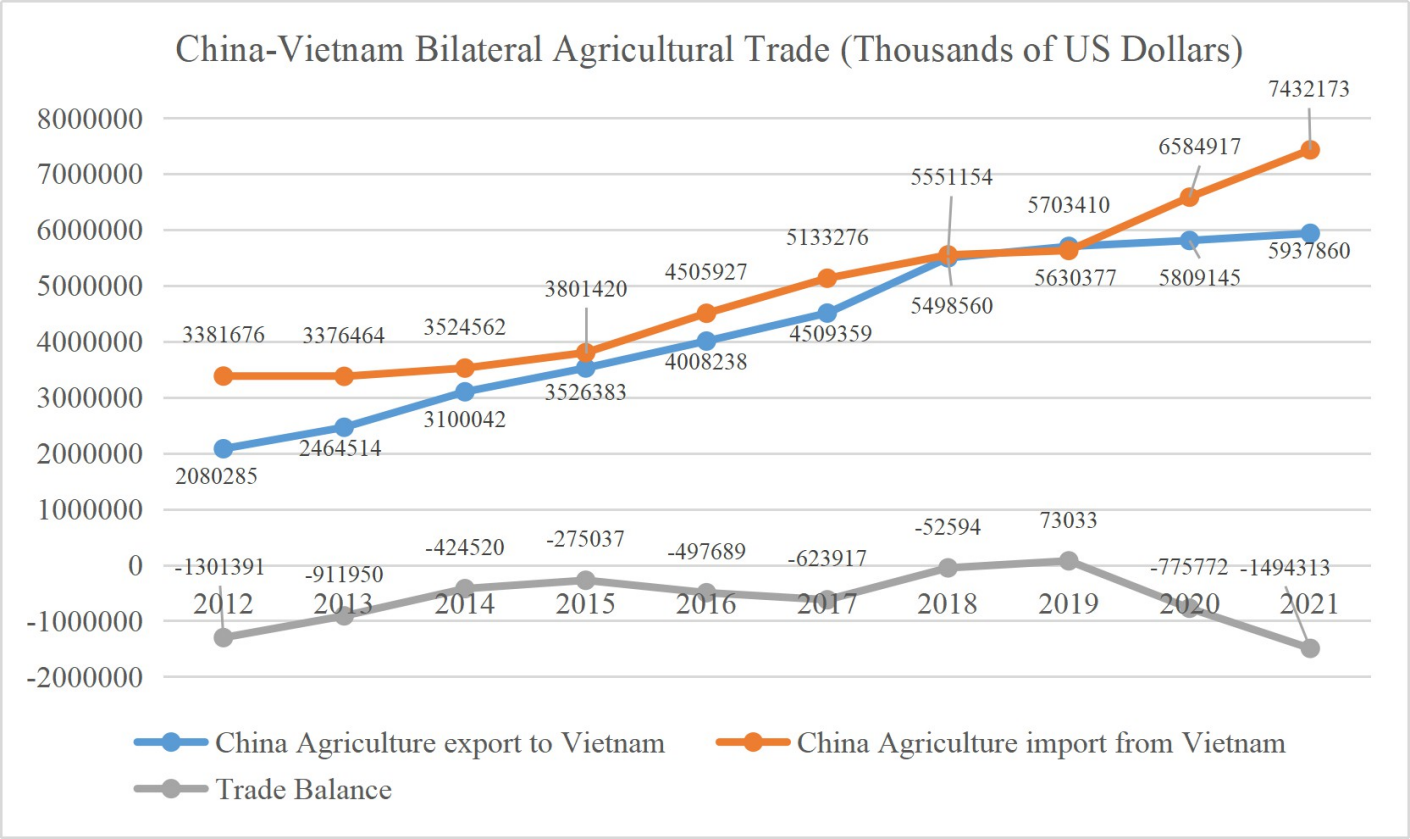
China-Vietnam bilateral agricultural trade volume between 2012 to 2021. Data Source: From United Nations Comtrade Database, 2022.

Data from the UN comtrade showed that (see Fig 1). China had a strong agriculture relationship with Vietnam. In 2021, China recorded a USD 1.49 billion deficit, the highest number recorded for an agricultural trade deficit between 2012 and 2021. China is working to boost its agricultural commodity imports from ASEAN nations.

From 2012 to 2021, China’s overall agricultural product trade showed an increasing trend with Vietnam, but the growth rate of exports was significantly lower than that of imports. Except for 2019, China’s trade with Vietnam’s agricultural products mainly had a deficit, and the growth rate was increasing yearly. Vietnam’s agricultural import and export trade grew steadily and significantly during this period, resulting in a surplus. The main export destinations of Vietnam mainly located in Asia, the United States, and Europe [5].

In light of these developments, recent studies have underscored the importance of analyzing competitive advantages and complementarity in agricultural product trade, particularly within China’s trade relations with other nations. Such as China and the countries along the “Belt and Road” [6, 7], Brazil, Canada, China, Sweden, Finland and the United States [8], China and India [9], Malaysia and ASEAN countries [10], Kazakhstan and China [11] and between China, Ghana and Myanmar [12, 13]. With growing competition in agricultural trade between China and Vietnam there is lack of studies between the two countries agri-trade, understanding their comparative advantages and complementarity becomes paramount. Such insights not only shed light on the intricacies of cross-border exports but also furnish valuable recommendations for enhancing trade the two countries trade relations [14].

This research endeavor seeks to delve into the dynamics of China and Vietnam’s bilateral trade in agricultural products, with a keen focus on assessing their comparative advantage and complementarity. By identifying barriers to economic growth in this sector, the study endeavors to furnish actionable recommendations aimed at fortifying international agricultural trade between the two nations.

## 2. Empirical literature and theoretical background

### 2.1 Comparative advantage and trade competitiveness of agricultural product trade

Competitiveness and complementarity are essential concepts in international trade. Comparative advantage theory and complementary trade theory are traditional economic theories that have been used to analyze trade effects in international trade sectors in recent studies.

Tao, analyzes the advantages and disadvantages of agricultural trade between China and Thailand from 2017 to 2019 [15]. Four methods including the Grubel-Lloyd index, Revealed comparative advantage index (RCA), Trade intensity index (TII), Trade complementarity index (TCI) were used to analyze the data. From the result, the Grubel-Lloyd Index indicated that Thailand has better competitive advantages than China overall in agricultural exports. The trade intensity index showed that the two countries have significant advantages in trading from better factors such as comparative advantage structures, better free trade agreements, and better geographical locations. The trade complementarity index also indicated that Thailand and China have specific complementary categories of agricultural products in one country’s export and another’s imports. The study found that Thailand and China have comparative advantages in different categories of agricultural products, and there is potential for cooperation in agricultural trade between the two countries. The study also found differences in the advantages of inter- and intra-industry trade for different agricultural products.

Imre Fertö and Hubbard [16], also analyze Hungary’s agricultural competitiveness with the EU from 1992 to 1998 using four revealed comparative advantage (RCA) indices. Results indicate Hungary has a comparative advantage in various agri-food products, including animals, meat, and cereals. The RCA indices, when interpreted as a binary measure, have remained relatively stable during the transition period, despite evidence of a weakening level of comparative advantage. These findings provide insights into underlying comparative advantage and implications for trade when Hungary joins the EU.

Zhou and Tong [6], examines the competitiveness of agricultural trade between China and countries along the Belt and Road initiative, using a sample of data from 2001 to 2019. They employ various indices to measure competitiveness, including market share, symmetry comparative advantage, revealed competitive advantage, and trade competitiveness. The results of their study show that the competitiveness of China’s agricultural products trade with Belt and Road countries varies across regions and over time. Key factors influencing competitiveness include the share of agricultural GDP, fertilizer input, capital stock, arable land area, and agricultural financial expenditure. These findings have implications for agricultural cooperation between China and countries along the Belt and Road.

Edjah, Wu and Tian [12] studied the agricultural trade competitiveness and complementarity between China and Ghana from 2016 to 2020. The study adopted the RCA and the TCI index. They found that China generally had a trade surplus, and the overall trade volume of agricultural products between the two countries had increased. China had a comparative advantage in certain categories, while Ghana had a comparative advantage in others. Analysis of trade complementarity showed potential for further cooperation.

### **2.2** The competitive advantages and complementarity of China-Vietnam agricultural product under China-ASEAN trade

The establishment of the Association of Southeast Asian Nations (ASEAN) Economic Community (AEC) in 2015 offers both opportunities and challenges to member countries. The main opportunities from AEC involve liberalizing trade in goods and services, protecting and promoting investment, narrowing down the social and economic development gap, and enhancing the free flow of skilled labor and freer flow of capital. In addition, ASEAN has signed free trade agreements with partner countries such as Australia and New Zealand, China, India, Japan, and Korea with various opportunities. Trade complementarity, rather than substitutability and competition, will drive the rapid increase of trade flows between the ASEAN parties.

Using the RCA, RTA, and NRCA indices, Hoang [17] analyzes the agricultural competitiveness of ASEAN countries. Findings showing that Vietnam, Thailand, and Indonesia have the strongest competitiveness, while Singapore and Brunei have the weakest. ASEAN countries are competitive in crop, wood, and fishery sectors, but there are differences between countries. Specialization in competitive products and regional integration can enhance competitiveness and social welfare.

Guo and Luo [18] study analyze the competitiveness and complementarity of China-Vietnam agricultural product trade from 2006 to 2016, with a view to providing reference for promoting the economic and trade cooperation between China and Vietnam. The paper adopted RCA index, export similarity index, and TCI index to analyze the competitiveness and complementarity of agricultural product trade between China and Vietnam. The authors also use revised intra-industry and intra-industry trade complementarity indices to study the reasons for the complementarity of China-Vietnam agricultural products. Their findings are that, the trade volume has increased, but China has been in a deficit state. The overall complementarity of the trade is relatively low, but there is a two-way trade relation between some agricultural products. China-Vietnam agricultural product trade is dominated by intra-industry trade and supplemented by inter-industry trade.

A study using Revealed comparative advantage (RCA) index and Relative Trade Advantage Index (RTA) indices by Ha, Liem, and Shuang [19], analyzed the export of rice between China and Vietnam. Trade volume was smaller and fluctuated more between these two countries than between China and other countries. China’s comparative advantage for exporting rice to Vietnam grew over time. Their results show that overall rice trade competition between China and Vietnam is not strong, which is conducive to improving trade cooperation and strengthening agricultural product cooperation.

## 3. Data description and methods

### 3.1 Description of data

The data are analysed and examined using the Trade Intensity (TI) Index, Trade Complementarity Analysis (TCI), and Revealed Comparative Advantage Index (RCA). The WTO classification of agricultural products is considered in this study with Vietnam, the commodity classification codes of the Standard International Trade Classification (SITC) Rev. 4 were used in this study to classify agricultural products. The SITC Rev.4 code in the United Nations database of trade-in goods statistics is listed in Table 1. As a sample size, the twenty (20) major agricultural product categories exported between China and Vietnam within ten years (from 2012 to 2021) were selected in this study. The results evaluated the complementarity and competitiveness of agricultural trade between the two economies and their current state of agricultural trade development in terms of trade policy, trade dependency, and trade structure.

**Table 1:** SITC Rev.4 categorization of agricultural product.

Section of SITC	code	Division of SITC
Food and Live Animals	00	Live animals
	01	Meat and meat preparation
	02	Dairy products and birds’ eggs
	03	Fish and fish product
	04	Cereals and cereals preparation
	05	Vegetables and Fruits
	06	Sugar, sugar preparation and honey
	07	Coffee, tea, cocoa, spices and manufactures thereof
	08	Feeding stuff for animals
	09	Miscellaneous edible products and preparations
Beverages and Tobacco	1	Beverages and Tobacco
Crude Materials inedible, except fuel	21	Hides, skins and fur skins, raw
	22	Oilseeds and oleaginous fruits
	23	Natural rubber
	24	Cork and wood
	26	Textile fibre
	29	Crude animal and vegetable materials
4. Animals and Vegetable Oils. Fat and Waxes	41	Animal oils and fats
	42	Fixed vegetable fats and oils, crude, refined or fractionated
	43	Animal or vegetable fats and oils, processed; waxes of animal or vegetable origin; inedible mixtures or preparations of animal or vegetable fats or oils

Data Source: From UN Comtrade Database.

### 3.2 Methods and materials

#### 3.2.1 Revealed Comparative Advantage (RCA) index

The Revealed Comparative Advantage index (RCA) was first presented by Balassa in 1965 [20]. According to a nation’s overall exports of commodities and worldwide exports of commodities, this index calculates the export ratio of a particular commodity [21] and recent studies such as [22, 23] adopted in their studies. The RCA index explains that, a country has a comparative advantage if the ratio is above 1. The RCA can be mathematically illustrated as:

$$RCA=\frac{X_{i\;j}^{k}/X_{i\;j}^{t}}{X_{i\;w}^{k}/X_{i\;w}^{t}}$$ (1)

Here RCA denotes the Revealed Comparative Advantage indicator;

$X_{i\;j}^{k}$ and $X_{i\;j}^{t}$ is the value of an agriculture product (k) that country (i) exports to country (j) and the total value of all goods that country (i) exports to country (j), respectively;

$X_{i\;w}^{k}$ and $X_{i\;w}^{t}$ is the export value of agriculture product (k) from country (i) that is exported to the world and the total quantity of all goods that country (i) exports to the world during the same time frame.

The RCA ranges of values between 0 and 1 (RCA<1) indicates a comparative disadvantage, while RCA greater than 1 (RCA >1) indicates a comparative advantage.

#### 3.2.2 Trade complementary index (TC)

The trade complementarity (TC) index is useful for predicting intraregional trade possibilities since it shows how closely a country’s import and export structures align. Furthermore, this index allows for a comparison of countries contemplating the development of a regional trade agreement to those who have already created or attempted to construct comparable arrangements. The TCI was improved by Drysdale [24], and current studies [12, 19, 25] used the TCI to measure how two countries’ agriculture export structures match with each other. The TCI between countries k and j is defined as follows:

$$C_{i\;j}^{k}={RCA}_{x\;i}^{k}\times {RCA}_{m\;j}^{k}$$ (2)

$C_{i\;j}^{k}$ is the trade complementarity index between the two nations i and j for agriculture commodity k;

${RCA}_{x\;i}^{k}$ represent the Export comparative advantage of country i in agriculture commodity k;

${RCA}_{m\;j}^{k}$ represent the Import comparative disadvantage of country j in agriculture commodity k;

${RCA}_{x\;i}^{k}$ and ${RCA}_{m\;j}^{k}$: Can be calculated as follow;

$${RCA}_{x\;i}^{k}=(\frac{X_{i\;w}^{k}}{X_{i\;w}^{t}})/(\frac{X_{w\;w}^{k}}{X_{w\;w}^{t}})$$ (3)

$${RCA}_{m\;j}^{k}=(\frac{M_{j\;w}^{k}}{M_{j\;w}^{t}})/(\frac{M_{w\;w}^{k}}{M_{w\;w}^{t}})$$ (4)

where $X_{i\;w}^{k}$ and $X_{i\;w}^{t}$ represent the trade volume of agricultural product k exported from country i to the world and the total agricultural export volume from country i to the world, respectively;

$X_{w\;w}^{k}$ and $X_{w\;w}^{t}$ represent the world export volume of agricultural product k and the world total agricultural export volume, respectively;

$M_{j\;w}^{k}$ and $M_{j\;w}^{t}$ represent the import volume of agricultural product k of country j from the world and the total agricultural import volume of country j, respectively; and

$M_{w\;w}^{k}$ and $M_{w\;w}^{t}$ represent the world import volume of product k and the world total agricultural import volume, respectively.

If country i possesses a relative advantage in producing commodity k, while country j lacks such an advantage, then it signifies that the two countries exhibit trade complementarity in commodity k. The extent of this complementarity can be assessed by analyzing their product $C_{i\;j}^{k}$. If $C_{i\;j}^{k}$ > 1, it indicates that the two(2) nations have trade complementarity in commodity k, and the higher the value, the greater the degrees of complementarity. If $C_{i\;j}^{k}$ <1, This shows that the level of complementarity is not significant, and the lower the value is, the lesser the extent of complementarity.

## 4. Result analysis and discussion

### 4.1 Result of Revealed Comparative Advantage (RCA)

The RCA measurement of agriculture trade between Vietnam and China was calculated in order to assess the competitiveness and comparative advantage of the two countries. The RCA Index values of the exports from both countries between 2012 and 2021 are presented in Table 2 below.

**Table 2:** China-Vietnam RCA of agricultural trade in products categories (2012–2021).

China					Vietnam			
Year	Category 0	Category 1	Category 2	Category 4	Category 0	Category 1	Category 2	Category 4
2012	0.47	0.08	0.29	0.05	8.79	2.07	1.00	0.37
2013	0.37	0.04	0.27	0.03	7.71	3.42	1.14	0.51
2014	0.35	0.04	0.23	0.02	6.73	3.77	0.89	0.41
2015	0.41	0.05	0.24	0.03	5.48	2.2	0.85	0.5
2016	0.46	0.06	0.28	0.06	5.32	1.43	0.73	0.18
2017	0.5	0.05	0.32	0.05	4.49	0.66	0.56	0.08
2018	0.53	0.05	0.3	0.06	3.52	0.73	0.51	0.06
2019	0.59	0.04	0.28	0.04	2.45	0.82	0.52	0.1
2020	0.59	0.06	0.22	0.03	1.15	0.49	3.29	0.72
2021	0.54	0.1	0.19	0.03	1.67	0.14	0.46	0.07

Data Source: calculation by the author based on data from the UN Commodity Trade Database.

China’s comparative advantage index is consistently low across all categories and years, as indicated in Table 2. From 2012 to 2021, the comparative advantage index consistently remains below 1. This is primarily attributed to China’s reform and development efforts, which have led to a shift in the country’s priorities towards industrialization and modernization. Consequently, there has been a decrease in the availability of arable land, prompting China to reduce its volume of agricultural exports in order to ensure food self-sufficiency. Vietnam’s RCA index exhibited a more pronounced comparative advantage in Category 0 and Category 1 throughout the designated period of 2012–2021, thus signifying its competitive edge in the Chinese market. However, after 2016, Vietnam’s agricultural products in Category 1 experienced a decline in their competitiveness, much like Category 2 after 2013. This result slightly deviates from Guo and Luo [18], but also in conformity with Ha et al. [19]. Moreover the result confirmed other related studies [26] stating that China has lost competitiveness in labor-intensive industries which includes the agricultural industry as this study result shows, while Vietnam’s competitive advantage in the agriculture trade is stronger than that of China’s.

Based on the aforementioned analysis, it can be inferred that Vietnam’s agricultural products exhibit higher competitiveness than those of China, despite the proximity, similar climate, and natural resource endowments of both countries. Additionally, a consideration of consumer demand reveals both similarities and disparities. Notably, China’s demand for differentiated products is more pronounced within its domestic market, this could be seen being less competitive in their agricultural product export compared with Vietnam. Furthermore, government policies play a significant role in shaping the competitive landscape.

### 4.2 Result trade complementary index

The study utilized the TCI methodology, to analyze and estimate the trade complementarity index of agricultural commodities between China and Vietnam. This analysis covered the period from 2012 to 2021, focusing on two measurement aspects: China’s exports to Vietnam and Vietnam’s exports to China. The findings indicate a strong trade complementarity between the agricultural products of China and Vietnam. Specifically, when considering China’s exports to Vietnam, category 0 agricultural products exhibit high complementarity indexes (TCI > 1). Similarly, when examining Vietnam’s exports to China, category 2 agricultural products show high complementarity indexes (TCI > 1) throughout the period from 2012 to 2021.

The trade complementarity index (TCI) between China and Vietnam is presented in Table 3. From 2012 to 2021, the results displayed that the Chinese-Vietnam export-import trade complementarity index is greater than 1 in category 0 of China’s Export (Vietnam’s Import) and category 2 of Vietnam’s Exports (China’s Import). This indicates that these categories of agricultural products complement each other in terms of import and export between the two countries. Others do not show a great deal of potential agricultural cooperation that exists between the two countries. Furthermore, this highlights that both countries have not fully utilized their resources and there exists room for efficient agricultural cooperation. Additionally, in recent years, both China and Vietnam have increased mutual investment, which could help expand the scale of internal trade between the two countries.

**Table 3:** The TCI of China–Vietnam agricultural product trade (2012–2021).

Measured with China’s Export (Vietnam’s Import)					Measured with Vietnam’s Export (China’s Import)			
Year	Category 0	Category 1	Category 2	Category 4	Category 0	Category 1	Category 2	Category 4
2012	1.14	0.13	0.47	0.12	0.00	0.08	1.55	0.27
2013	1.14	0.13	0.45	0.12	0.01	0.09	2.22	0.23
2014	1.08	0.12	0.52	0.13	0.05	0.09	1.91	0.19
2015	1.14	0.13	0.51	0.12	1.82	0.11	1.98	0.19
2016	1.21	0.13	0.47	0.1	0.43	0.10	2.27	0.15
2017	1.17	0.13	0.47	0.11	0.51	0.10	2.26	0.57
2018	1.17	0.13	0.52	0.15	0.09	0.09	2.25	0.10
2019	1.16	0.11	0.53	0.18	0.36	0.10	1.77	0.20
2020	1.19	0.09	0.50	0.22	0.29	0.06	2.84	0.14
2021	1.21	0.08	0.51	0.23	0.41	0.06	2.21	0.16

Data Source: calculated by the author according to data from the UN Commodity Trade Database.

## 5. Conclusion and suggestions

### 5.1 Conclusion

This paper evaluated the complementarity and competitiveness of China-Vietnam’s agricultural product trade. It is evident that both China and Vietnam have a strong trade relation in agriculture product. Thus, there is frequent exchange of agricultural products between the two countries and China highly depends on Vietnam’s agriculture products. Furthermore, when examining the comparative advantage and complementarity of the countries’ agricultural product trade, using the RCA index analysis, Vietnam has stronger comparative advantages than China over the selected period of the study. In contrast, they both have complementarity in particular agricultural product categories according to the TCI index analysis. Cooperation between China and Vietnam in agricultural trade has potential benefits. Nonetheless, this study has few limitations; 10-year data analysis can only be used short-term, whereas long-period data may introduce more deeper analysis result and understanding. Additionally, future studies could further could empirically examine the factors influencing these results, specifically future studies could explore the implications of exchange rate fluctuations on trade competitiveness, pricing strategies, and market dynamics in greater detail. Agriculture is one of Vietnam’s most important exports, significantly increasing total export turnover. Therefore, following an evaluation of the current scenario in conjunction with an analysis of the comparative advantage and complementarity of agriculture-related trade between China and Vietnam, this article proposes suggestions for the continuation and initiation of agricultural trade collaboration between the two nations.

### 5.2 Suggested recommendations

#### 5.2.1 Impose efficient Policy and bilateral trade agreement

China and Vietnam should effectively combine policies such as offering zero-tariff treatment for current agricultural products, price policies, export policies, etc., to boost agricultural exports in international integration. The two nations should have protective policies and special programs to support domestic export enterprises while adhering strictly to WTO regulations. Furthermore, China and Vietnam are members of the Regional Comprehensive Economic Partnership (RCEP), a free trade agreement of the next generation with broader and deeper commitments. It is recommended that Vietnam aligns the rules of origin within the RCEP agreement to enhance the competitiveness of its exports in RCEP markets, particularly China, instead of relying solely on the ACFTA. The RCEP agreement provides an opportunity to establish a comprehensive framework for trade policy, investment, intellectual property, e-commerce, and dispute resolution, fostering fair trade practices and unlocking potential for regional industries, including China’s agriculture sector, to compete effectively.

#### 5.2.2 Optimize and adjust the structure of China-Vietnam agricultural trade

China has a trade deficit in agricultural product trade with Vietnam. This results from an imbalance between the demand and supply of domestic agricultural products in China. The "Belt and Road" initiative has reduced trade expenses between China and the countries along the route, leading to increased imports of agricultural products from Belt and Road Initiative (BRI) countries. To address this trade deficit between China and Vietnam, it is necessary to revamp the trade framework and strive for balanced bilateral trade. In addition, both countries could also specialize in producing and exporting agricultural goods with comparative advantages while importing uncompetitive products to boost regional trades, effectively utilize their economic resources, and increase social welfare by eliminating their trade restrictions and reducing production subsidies.

#### 5.2.3 Develop markets and international economic integration

Both countries should promote agro-processing chains alongside the development of retail systems, the creation of brands for essential national products, and the marketing of regional specialties with associated geographical indications. In addition, in collaboration with the Trade and Industry Ministry, the Foreign Affairs Ministry, local governments, industry associations, businesses, and farmers expand export markets, bolster market forecasting capabilities, and promote trade promotion. The government should assist businesses with legal issues to minimize international integration risks.

#### 5.2.4 Integrate bilateral agricultural market information

The information system should be completed with information on the business environment, distribution system, domestic and foreign agro-product prices, and short-term and long-term projections, allowing people and entrepreneurs access to accessible information [27]. Two-way information routes connecting leaders to producers, merchants, and customers should be established. Market information should be widely disseminated; industrial organization, output targets, commercial promotion tactics, and crop forecasting should be modernized, with a strong interaction between the public and private sectors.

Building a contingent for market knowledge and forecasting should be prioritized. Market information activities should be organized regularly (conferences on industry forecasts, market news, market TV and radio channels, etc.); information should be delivered to farmers and firms throughout the value chain in a timely, comprehensive, and accurate manner.

Investment should be undertaken in establishing agro-product trading platforms that connect strategically specialized areas with commercial activity in key international markets. Risk management measures should be implemented following the market mechanism, such as insurance, the construction of warehouse systems, the use of modern transaction forms (bidding, scheduled delivery, e-commerce, etc.), reducing risks to a bare minimum, and avoiding risks caused by market fluctuations.

#### 5.2.5 Adoption of traceability technology

To meet the standards for origin, production, and processing required for Vietnamese agricultural products, especially those destined for the Chinese market, it is crucial to incorporate traceability technology. By integrating traceability technology into every phase of product development, Vietnamese agricultural products can expand their market reach. Given the growing demand for transparency from consumers, traceability is considered the optimal solution and an inevitable trend for Vietnamese products. When traceability technology is implemented, customers can access comprehensive information about the various stages of production and distribution, from the source to the final product. Currently, manufacturing companies prefer to use information technology and electronic devices for data management, updates, and product traceability. The strict traceability requirements in the Chinese market present Vietnamese enterprises with an opportunity to establish a strong presence and enhance the reputation of Vietnamese goods in the international market.
